# The contribution of case study design to supporting research on Clubhouse psychosocial rehabilitation

**DOI:** 10.1186/s13104-015-1521-1

**Published:** 2015-10-01

**Authors:** Toby Raeburn, Virginia Schmied, Catherine Hungerford, Michelle Cleary

**Affiliations:** School of Nursing and Midwifery, Western Sydney University, Locked Bag 1797, Penrith, NSW 2751 Australia; School of Nursing, Midwifery, and Indigenous Health, Faculty of Science, Charles Sturt University, Wagga Wagga, NSW Australia

**Keywords:** Case study, Clubhouse, Design, Mental health, Recovery, Research

## Abstract

**Background:**

Psychosocial Clubhouses provide recovery-focused psychosocial rehabilitation to people with serious mental illness at over 300 sites in more than 30 countries worldwide. To deliver the services involved, Clubhouses employ a complex mix of theory, programs and relationships, with this complexity presenting a number of challenges to those undertaking Clubhouse research. This paper provides an overview of the usefulness of case study designs for Clubhouse researchers; and suggests ways in which the evaluation of Clubhouse models can be facilitated.

**Results:**

The paper begins by providing a brief explanation of the Clubhouse model of psychosocial rehabilitation, and the need for ongoing evaluation of the services delivered. This explanation is followed by an introduction to case study design, with consideration given to the way in which case studies have been used in past Clubhouse research. It is posited that case study design provides a methodological framework that supports the analysis of either quantitative, qualitative or a mixture of both types of data to investigate complex phenomena in their everyday contexts, and thereby support the development of theory. As such, case study approaches to research are well suited to the Clubhouse environment. The paper concludes with recommendations for future Clubhouse researchers who choose to employ a case study design.

**Conclusions:**

While the quality of case study research that explores Clubhouses has been variable in the past, if applied in a diligent manner, case study design has a valuable contribution to make in future Clubhouse research.

## Background

Established towards the end of the 1940′s, the Clubhouse model is one of the world’s oldest approaches to psychosocial rehabilitation [1]. Popular worldwide, there are currently over 300 Clubhouses operating in more than 30 countries [2]. People who attend Clubhouses typically have a history of serious mental illness and face a number of challenges, including those related to their physical health, social welfare and employment [3]. In response, Clubhouses provide a wide range of social, health, educational and employment support programs [2]. To encourage a sense of empowerment and belonging, participants in these programs are referred to as ‘members’ rather than ‘patients’ or ‘consumers’ [4].

Clubhouse members follow an activity schedule referred to as the ‘work ordered day’ [5], where they work alongside paid staff, often assuming lead roles and taking responsibility for all aspects involved in running the Clubhouse. By contributing in these proactive ways, members embrace opportunities to build confidence, friendships and skills, while also being encouraged to pursue educational and employment goals in the wider society [6]. Building on these activities, Clubhouse programs referred to as Transitional Employment Programs (TEP) are then tailored to support members who decide to seek work in the competitive job market [6].

Clubhouses have been at the forefront of advocacy for consumer centred, recovery-oriented practice [7, 8]. Despite this, researching the complex nature of these services has proved challenging [9, 10]. Clubhouse research is further complicated by the highly personalised and context-dependent ways that people experience mental health recovery [11]. Reflection on such challenges has led to long consideration of the research design that best supports the exploration and explanation of the way in which Clubhouses work to support recovery—that is, the ‘recovery orientation’ of the Clubhouse model [12]. One research method with the potential to provide a rigorous framework for exploring phenomena within organisations such as the Clubhouse is case study design [13, 14].

Case study design typically uses multiple perspectives to facilitate the examination of a particular phenomenon in its natural context [15, 16]. While this may sound similar to the goal of many qualitative research approaches, case study design is different because it can be flexibly adapted as a framework that incorporates either qualitative, quantitative or a mixture of qualitative and quantitative research approaches [13]. Case study design is also unconstrained by a particular theoretical approach, meaning it can be pragmatically informed by or used to build or critique any theory related to the phenomena in question [17].

According to Tight [18], publications on the topic of case study from the past decade have been dominated by the work of two leading theorists, Yin [16] and Stake [19]. Yin [16] divides case studies into two broad groups. First, those that focus on an individual case, involving detailed exploration of either a person or an organisation. These are referred to as a ‘single case study’. Second, those that involve investigation of a group of cases for comparison and contrast are referred to as ‘multiple case studies’. Yin then makes a further division, categorising each case study as either exploratory, descriptive or explanatory.

Exploratory case studies are commonly pilot projects that seek to reveal what phenomena or theory exists within a field of interest. For example, a researcher interested in how services assist people with mental illness to achieve recovery, may seek to discover if there are any guiding recovery principals used by mental health services. Such a study may uncover phenomena and/or theory that can then lead to further investigation.

In contrast, descriptive case studies begin with a theory about a phenomena, and then seek to chronicle how the phenomena is displayed through the lens of those theoretical assumptions. For example, a descriptive study may set out to elucidate how certain recovery principles are reflected in the practices of a Clubhouse. A risk with this type of case study is that the researcher may find that the theory brought to the project is not applicable which, in turn, may lead to the need for further exploratory work.

Finally, explanatory case studies seek to interpret why a particular phenomenon or theory has been revealed in the data. This approach is cited as being particularly useful in a multiple case study design, because pattern-matching can be used. For example, a study may seek to explain why work seems to be important to the rehabilitation of people with mental illness at three different Clubhouses located across a variety of cultural contexts [16].

For Stake [19], case study design is focused on the exploration of a case and refining or revealing related concepts. Stake [19] divides case studies into intrinsic, instrumental or collective designs. Intrinsic design is used when researchers have a particular interest in improving their understanding of a phenomenon. This method is described as being primarily aimed at exploring rather than understanding theoretical constructs. In contrast, instrumental design refers to those case studies that seek to elucidate phenomena and test or strengthen theory. With this approach, the case and its context are studied in depth to facilitate deep understanding of a concept. Finally, collective case studies include any study involving more than one case, similar to Yin’s [16] description of ‘multiple case design’.

Consideration of the explanations provided by Yin [16] and Stake [19] suggest that case study may be described as a flexible research design that may utilize either qualitative, quantitative or a mixture of both types of data, to illuminate, elucidate or interpret phenomena in their everyday context and support the development of theory. This definition is important in this paper because it provides a framework for considering case study design in relation to Clubhouse research. For example, while several studies have described people’s subjective experience of recovery in psychosocial Clubhouses [11], there has been limited research exploring the way Clubhouses implement recovery-oriented practices. In this paper we review how case study research has contributed to the field of Clubhouse psychosocial rehabilitation.

## Method

Initially, this paper was conceived as an integrative literature review that examined the published case studies that have contributed to Clubhouse research. An electronic literature search was conducted seeking to identify full text peer reviewed journal articles written in English and published between 1960 and January 2015. The papers were required to refer to themselves as a ‘case study’ or derivative, and to have a focus on a Clubhouse or Fountain House. The search term ‘Fountain House’ was included because, as the name of the original Clubhouse, this term is popular in Clubhouse related literature.

The search terms, “case stud*” AND “clubhouse” OR “fountain house” were combined across three databases, leading to initial identification of 41 papers from PsycINFO, 20 from CINAHL and 16 from Proquest Social Science Journals. Reference lists were checked for other relevant papers, then following article screening and removal of duplicates, five papers were identified as relevant to the review [20–24]. All based in North America, the five articles were all published more than a decade ago, with one published as early as 1960.

The quality of each paper was initially assessed by the Chief Investigator (TR), using the Critical Skills Appraisal Program (CASP) [25]. CASP posits there are three broad issues that should be considered when appraising qualitative research, these are;

- Are study results valid?
- What are the results?
- Will the results help locally? [25]

A ten question, three point scale was used to assess for validity, results and relevance. CASP ratings and notes were reviewed by all authors. The assessment was problematic however, as the majority of papers identified had been published in an era when diligent approaches to case study research and reporting (such as ethics approval) were often not applied. The consensus view amongst the authors was that this small sample of case studies could not bear the scrutiny of modern analytical techniques as part of an integrative literature review. Despite this, the results did provide useful information regarding the use of case study design in Clubhouse research, including the advantages and disadvantages. In turn, this prompts a variety of considerations for researchers who may consider using case study design in Clubhouse settings in future, with these considerations outlined in the results and discussion section presented below.

## Results and discussion

### Advantages and disadvantages of case study design in Clubhouse research

In common with qualitative research approaches such as ethnography, an emphasis on studying phenomena in its natural context means case study design incorporates the perspectives of participants who may come from vulnerable and voiceless groups in society [26]. For this reason, case studies have often been used to provide a framework to critique oppression and question social norms [27]. This suggestion was exemplified in the earliest evidence of a published Clubhouse case study, a paper by Goertzel et al. [22] published in 1960 that described the original Clubhouse in New York City during its early development. Using multiple data sources, the paper provided a rich description of the theoretical orientation, history, facilities, staff, volunteers, membership and programs available [22]. The research is important because it was written in an era when society held stigmatizing attitudes towards people with serious mental illness, who often spent their lives in custodial psychiatric institutions [28, 29]. The paper by Goertzel et al. [22] conveyed ideas ahead of its time regarding the importance of involving people with a lived experience of mental illness in the development and delivery of mental health services. This case study, then, provides evidence of the early role that Clubhouses played in advocating for recovery-oriented models of mental health care.

Another advantage of case study design is the way in which it can be flexibly adapted to incorporate a mixture of qualitative and quantitative methods, as promoted by researchers such as Creswell [26, 30]. An example of a mixed methods case study was conducted by Boll [20], who undertook a case study of a Clubhouse in New Jersey to explore the phenomena of empowerment among Clubhouse members involved in a service evaluation. Using a combination of quantitative and qualitative data collection methods, including survey questionnaires, participant observation, and individual interviews, the study found that researching Clubhouse members within the regular Clubhouse environment led to benefits such as enhanced engagement with new members and improved program quality [20].

A final advantage of undertaking case study research relates to the way in which it can support the testing of connections between theory and phenomena [31]. This characteristic was demonstrated in a Clubhouse case study conducted by Cowell et al. [24]. The study explored the concept of ‘function cost’, a theory designed to explain the financial cost to services that utilize co-production, where consumers are involved in both delivery and receipt of services. The boundaries in the study were difficult to ascertain because Clubhouse members were involved in the provision of tasks normally delivered by paid staff in hospital-based services. The researchers addressed this dilemma pragmatically by using two standardised research scales to collect separate financial data about costs associated with paid staff and voluntary labour invested in activities. Results from the study suggested that the concept of ‘function cost’ may provide a way to explain the financial costs of Clubhouse programs utilising co-production practices [24].

As is evident from the above examples drawn from Clubhouse research, there is no standardised way to apply case study design. Instead, this flexible approach offers researchers the opportunity to select from a variety of methods and data collection techniques to ensure a ‘best fit’ for the case in question. As with any style of research however, case study design also has some disadvantages.

One of the most commonly cited disadvantages of case studies is that findings can lack generalizability [15, 16]. This suggestion, along with arguments that case studies lack scientific credibility because replication is difficult, has led to research regulators such as Australia’s National Health and Medical Research Council (NHMRC) [32] ranking case study as the lowest form of credible research design. Following scientific convention, the NHMRC [32] has ranked the quality of the designs of research, with some designs posited as producing more rigorous evidence than other research designs. For example when evaluating the effectiveness of an intervention, a Randomised Controlled Trial (RCT) is regarded as providing the most reliable evidence [33].

The NHMRC [32] suggests that the processes integral to RCTs minimize the risk of confounding factors and highlight that internal validity is generally stronger in randomized control trials. However external validity can be stronger in multiple case study designs, and can be weak in randomized control trials. Such weaknesses in RCT design have been exposed in a number of systematic reviews and secondary analyses. For example, Hunt, Siegfried, Morley, Sitharthan and Cleary [34] completed a Cochrane review of psychosocial interventions for people with serious mental illness examining 32 RCTs. Contrary to the view that RCTs provide a rigorous, dependable research design, the authors reported substantial difficulties with skewed data, risk of bias, poor trial methods, small sample sizes, low event rates and wide confidence intervals [34]. In another example related to Clubhouse employment programs, Johnsen et al. [35] conducted a secondary analysis of a multisite RCT and found that a limited definition of ‘competitive employment’ and variability in ‘control’ conditions, across sites, led to skewed findings. Johnsen et al. [35, 36] together with other researchers, have gone on to observe that these kinds of variation in definition and control conditions in RCTs have led to substantial inconsistencies in research of employment services for people with serious mental illness.

Responding to criticism of case study design, theorists such as Yin [16] have suggested that generalisation of findings from case studies should focus on assessing the efficacy of theoretical constructs, rather than on the transferability of statistics. As mentioned previously, such a focus on theoretical concepts was exemplified in a case study by Cowell et al. [24], which explored the usefulness of the ‘function cost’ concept. Stake [19] has also argued that case study findings can be transferable, but from a different point of view. He suggests that readers can normally relate to the findings of case studies, which facilitate a kind of generalised understanding of phenomena [19]. For example, Jacobs used a case study design to provide an illuminating description of the challenges associated with improving access to psychiatry for members at a Clubhouse [23].

In contrast to his strong advocacy for the efficacy of case study design, one disadvantage observed by Yin [37] is that case study researchers can lack discipline, sometimes allowing detailed description and illustrative quotes to dominate findings. According to Yin, this is often at the expense of detailed accounts of research design procedures such as ethics, data collection and analytic procedures. An interesting technical point consistent across the five papers identified in this review was the lack of clarity regarding ethics and consent [20–24]. For example, Asmussen et al. [21] completed an interesting case study of a Clubhouse outreach program for homeless people, but failed to include any reference to ethical considerations.

In an effort to promote quality case study research, theorists such as Feagin [38], Yin [16] and Stake [39] have sought to develop protocols and structures for applying case studies. The following section will outline some considerations for effective application of case study design in future Clubhouse research.

### Considerations for conducting case studies in Clubhouse settings

Assuming that a research question has been identified and that the researchers’ choice of case study design is driven by a desire to explore a phenomenon in depth in its everyday context, the next logical step is to identify whether the case best fits a single or multiple case design [16]. Single-case design may be a suitable choice if the case displays particular uniqueness—for example, a study into the unique cultural experience of needing to ‘save face’ experienced by members of a Hong Kong Clubhouse [40]; or the development of an innovative program integrating a psychiatry clinic into a Clubhouse [41]. A single case approach may also be useful for a study that has limited time and access to resources, such as a student undertaking higher degree studies that involve a research project. It is important at the outset that the researcher is clear about how findings will be analysed, and compared to or tested against a theoretical paradigm [19].

Alternatively, multiple-case design may work well in situations where there are several similar cases that can provide pathways for replication and comparison [39]. Replicating a case study in this way would then present the opportunity for pattern-matching, a technique that links several pieces of information from the same case to a theoretical proposition, thereby enhancing the rigour of findings and generalizability of theory [42]. For example, research providing theoretical observations about the Clubhouse’s supported employment programs might be strengthened by using a multiple case study design that includes Clubhouses of different sizes across a variety of cultures. This could then potentially enable generalisation of findings to the Clubhouse model as a whole.

Following the identification of whether a single or multiple case study is best suited to a research question, Yin [16] contends that a structured approach to design should be taken to ensure quality and exploratory power in case study research. He suggests that case study design should include:

- An overview of the case study project citing objectives, issues and background.
- Written field procedures describing research location and access to data.
- Identification of research questions to be focused on during data collection.
- A reporting guide outlining a general format for the report.

By employing such points as a guide, then, researchers will support consistency across case study research undertaken in a Clubhouse context.

Common data sources include but are not limited to, documentation, archival records, interviews, direct observation, participant observation and physical objects [16]. While no individual source should be considered better than another, the rationale for using several sources of data is the triangulation of evidence. Triangulation provides checks and balances for the reliability of data collection [43]. For example, data drawn from participant observation and interviews could be used to corroborate the meaning and application of data revealed through review of a Clubhouse’s documentation.

Conducting research in any service for people living with mental illness requires special sensitivity [44]. To encourage empowerment and guard against any potential harm to participants the Clubhouse model has a strong commitment to the co-production of research with members regularly encouraged to ask questions and share points of view [45]. With this in mind, a collaborative approach should be planned, actioned and reflected upon when conducting any Clubhouse case study.

A further consideration is promoting quality mental health research. People with serious mental illness often experience stigma and marginalization, and so it is important that research does not perpetuate this [44]. Developing a strong evidence base is crucial however, and within fields of mental health research there is robust debate regarding the merits and weaknesses of the different research paradigms [44]. Regardless of what approach is taken, consumers must be positioned at the centre of any mental health research—and genuine consultation with stakeholders is essential, including respectful processes, as well as ethical behaviours and practices, to ensure that research contributes to the nature, quality and the validity of the data gained [46].

Evaluation of case study designs may be conducted in a number of ways. As mentioned previously, the CASP [25] provides a ten point tool for systematic consideration of study design, results, validity and relevance. Alternatively, Popay’s [47] method of appraisal places a high value on studies that validate the expertise of consumers of healthcare and the theoretical generalizability of findings. Using this appraisal method, the research is rated as ‘thin’ if there is little consideration of consumer insights, limited explanation, and low relevance for generalization. On the other hand, studies are considered ‘thick’ if they lend weight to consumer descriptions, including detailed description of phenomena; and show potential for generalizability [48]. Much of the data found in older Clubhouse research, struggles to find relevance when tools like CASP [25] and Popay’s [47] approach are applied. While this does not diminish the value of early research, as the Clubhouse model continues to evolve, appraisal tools may provide substantial benefit for evaluating and improving the quality of modern Clubhouse case studies.

## Conclusion

Psychosocial Clubhouses serve some of the most vulnerable and marginalised people in society. The Clubhouse model has become an internationally regarded provider of consumer-centred recovery-focused psychosocial rehabilitation [7, 11, 49]. With these considerations in mind, there is high need for research designs capable of exploring and describing how Clubhouses implement recovery practices.

This paper has identified case study design as a flexible research design that may utilize either qualitative, quantitative or a mixture of both types of data, to illuminate, elucidate or interpret phenomena in their everyday context and support the development of theory. As health science continues to evolve, case study design can provide a flexible framework for exploring the complex challenges presented by multidimensional mental health services like Clubhouses. Case study design enables consumers to play a central role in the development, implementation, analysis and synthesis of research. It also supports the conduct of genuine consultation with stakeholders, including respectful processes, ethical behaviours and practices to ensure the quality and validity of data gained.

## Abbreviations

CASP: Critical Skills Appraisal Program
NHMRC: Australia’s National Health and Medical Research Council
RCT: randomised controlled trial
